# Osteogenic cell response to 3-D hydroxyapatite scaffolds developed via replication of natural marine sponges

**DOI:** 10.1007/s10856-015-5630-0

**Published:** 2015-12-24

**Authors:** S. A. Clarke, S. Y. Choi, Melanie McKechnie, G. Burke, N. Dunne, G. Walker, E. Cunningham, F. Buchanan

**Affiliations:** School of Nursing and Midwifery, Queen’s University of Belfast, Medical Biology Centre, 97, Lisburn Road, Belfast, BT9 7BL UK; School of Mechanical and Aerospace Engineering, Queen’s University of Belfast, Ashby Building, 121 Stranmillis Road, Belfast, BT9 5AH UK; School of Biological Sciences, Queen’s University of Belfast, Medical Biology Centre, 97, Lisburn Road, Belfast, BT9 7BL UK; Engineering Research Institute, School of Engineering, Ulster University, Jordanstown Campus, Shore Rd, Newtownabbey, BT37 0QB UK; School of Mechanical and Manufacturing Engineering, Dublin City University, Glasnevin, Dublin, 9 Ireland

## Abstract

Bone tissue engineering may provide an alternative to autograft, however scaffold optimisation is required to maximize bone ingrowth. In designing scaffolds, pore architecture is important and there is evidence that cells prefer a degree of non-uniformity. The aim of this study was to compare scaffolds derived from a natural porous marine sponge (*Spongia agaricina)* with unique architecture to those derived from a synthetic polyurethane foam. Hydroxyapatite scaffolds of 1 cm^3^ were prepared via ceramic infiltration of a marine sponge and a polyurethane (PU) foam. Human foetal osteoblasts (hFOB) were seeded at 1 × 10^5^ cells/scaffold for up to 14 days. Cytotoxicity, cell number, morphology and differentiation were investigated. PU-derived scaffolds had 84–91 % porosity and 99.99 % pore interconnectivity. In comparison marine sponge-derived scaffolds had 56–61 % porosity and 99.9 % pore interconnectivity. hFOB studies showed that a greater number of cells were found on marine sponge-derived scaffolds at than on the PU scaffold but there was no significant difference in cell differentiation. X-ray diffraction and inductively coupled plasma mass spectrometry showed that Si ions were released from the marine-derived scaffold. In summary, three dimensional porous constructs have been manufactured that support cell attachment, proliferation and differentiation but significantly more cells were seen on marine-derived scaffolds. This could be due both to the chemistry and pore architecture of the scaffolds with an additional biological stimulus from presence of Si ions. Further in vivo tests in orthotopic models are required but this marine-derived scaffold shows promise for applications in bone tissue engineering.

## Introduction

Current clinical strategies for bone repair have accepted limitations, such as adequate donor site morbidity, adequate supply and concerns about disease transmission [1–3]. Synthetic bone graft materials are commercially available but often their use in the clinic is limited as surgeons are concerned by poor or variable clinical outcomes. The criteria required of a bone graft material are many and varied as they may need to support bone growth in a mechanically loaded environment. Current opinion would suggest that the material should be non-inflammatory, osteoconductive, bioactive, bioresorbable, porous and have a degree of mechanical strength [4].

The inclusion of porosity in this list created a dichotomy between mechanical strength and percentage porosity and sparked huge debate over optimum pore features; pore size, connectivity of the pores (i.e. whether they are open channels connecting to others or closed cul-de-sacs), their tortuosity (the “difficulty” of the route through the material) and the overall porosity. Decades after Hulbert and Klawitter suggested the use of porous structures to improve bone integration into synthetic materials [5, 6], there remains little consensus on the optimum pore size, either for resorption or bone ingrowth [7], with suggestions ranging from mean pore sizes of 100 μm to as large as 500 μm diameter [8]. More recently, the addition of microporosity (<10 μm) has been shown to enhance bone repair [9, 10] perhaps by improving fluid flow and promoting neovascularization [11].

In truth, there is probably no single optimum pore size and a range of pore diameters and pore features may allow the material to function on a number of different levels. Biomimetic strategies have led to the investigation of naturally occurring porous structures as templates for bone growth and the marine environment, in particular, is rich in mineralizing organisms with porous structures, some of which are currently being used as bone graft materials and others that are in early stages of development [12]. The naturally occurring interconnectivity, pore size distribution and tortuosity provide unique templates for material design that cannot be produced using current manufacturing techniques replicated synthetically [13].

The authors have previously demonstrated the ability to produce 3-D porous hydroxyapatite scaffolds from marine sponge templates which preserve the porous architecture of the organism [14, 15]. These scaffolds have both microporosity and macroporosity with 99.9 % interconnectivity, good permeability and improved mechanical properties when compared to scaffolds derived from a synthetic polymer foam [16]. The aim of the current study was to assess the ability of the marine-derived scaffolds to support osteoblast-like cell proliferation and differentiation in vitro when compared to synthetic polymer-derived scaffolds with the hypothesis that the naturally occurring pore architecture of the marine-derived scaffolds would better support cell growth and infiltration.

## Materials and methods

### Cell preparation

Two cell types were used for this study, human foetal osteoblast cell line (hFOBs; LGC Standards, USA) and primary guinea pig bone marrow stromal cells (gpBMSCs). gpBMSCs were isolated from whole bone marrow following sacrifice. Briefly, both tibia and femora were dissected out and cleaned of soft tissue. The ends of each bone were removed and marrow flushed out with sterile phosphate buffered saline (PBS). Following centrifugation, the cell pellet was re-suspended in 8 mL of PBS, layered onto 4 mL of Lymphoprep (Sigma Aldrich, UK) and centrifuged at 800 g for 40 min. The buffy layer was removed, washed and re-suspended in complete medium [DMEM supplemented with 20 % FBS + 2 mM l-glutamine + 100 U/mL Pen/strep + 2.5 μg/mL fungizone (all Gibco from Life Technologies, UK)] for counting. Cells were plated at 1 × 10^5^ cells/cm^2^, left undisturbed for 7 days and then fed biweekly thereafter. Cells were passaged (1 flask: 4 flasks) when approximately 90 % confluent using 0.25 % trypsin/EDTA (Gibco).

### Scaffold preparation

**Fig. 1:**
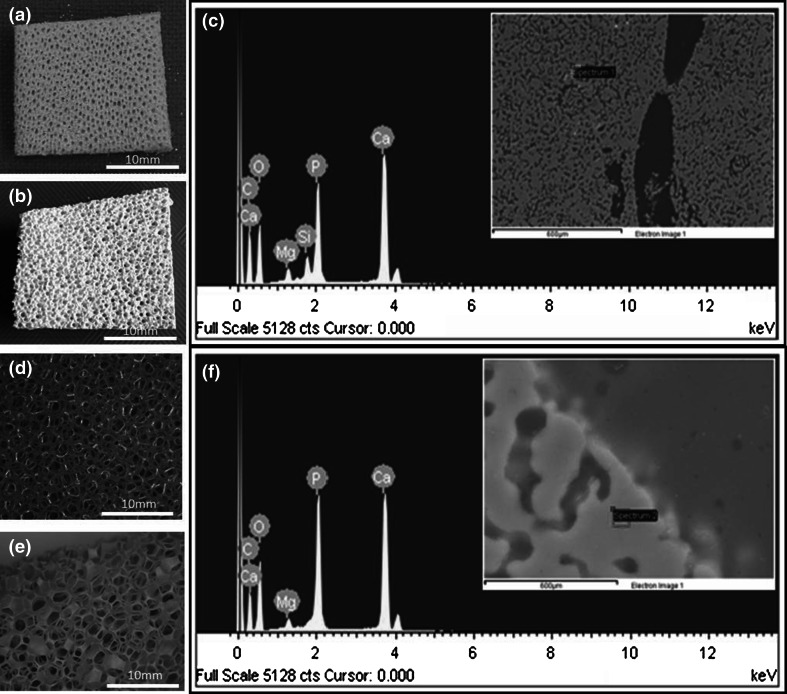
Gross images of *Spongia agaricina* (**a**) and polyurethane sponge (**d**) templates before processing and HA marine sponge-derived (**b**) and PU sponge-derived scaffolds (**e**) following replication. EDX elemental analysis of HA scaffolds (**c** marine derived; **f** PU derived) showing the presence of Mg (both scaffolds) and Si (marine-derived)

Scaffolds approximately 10 mm in diameter and 10 mm in length were prepared of each material (Fig. 1). Ceramic slip [14] and scaffold production [15] have been reported in previous publications. Briefly, predominantly spherically shaped particles of HA grade S-BM (Batch P260/S/BM/192; Plasma Biotal Ltd, UK) between 820 nm and 16.2 μm were mixed with 2 wt% ammonium polyacrylate (Darvan 821A; R.T Vanderbilt Company, USA), an anionic polyelectrolyte. Through the addition of ever decreasing amounts of HA powder to a mixture of distilled water and Darvan 821A over a period of 4 days, an 80 wt% (55.9 vol%) solid loaded slip with a viscosity of 126 mPas was achieved.

Scaffold production involved submerging the flexible polyurethane (PU) packaging foam (∅10 mm, 10 mm height; 60 pores/in2; density, 30 kg/m3) (Craftworld Ltd, UK) and the marine sponge (10 × 10 × 7^−12^ mm), *Spongia agaricina* (Pure Sponge UK Ltd, UK), in the optimized 80 wt% HA slurry followed by squeezing in a Collin W100T Two Roll Mill (LRS Planung and Technologie GMBH, Germany). The specimens were dried for 4 h and sintered in a box furnace (EliteThermal Systems Ltd, UK) at 1300 °C. A ramp rate of 5 °C min^−1^, a cooling rate of 3 °C min^−1^, and a dwell time of 5 h were determined as the optimal sintering regime.

Using the theoretical density of fully densified polycrystalline HA (3.156 g cm^−3^), the relative density was calculated for five scaffolds and an average porosity value was obtained. Table 1 confirms that the pore characteristics of the replicated scaffolds are similar to those previously found. Elemental analysis was assessed using scanning electron microscopy (SEM) and energy-dispersive X-ray spectroscopy (EDX) at 15 kV accelerating voltage under medium probe current (JEOL 6500F, JEOL Ltd., Japan). Scaffolds were washed ×3 in PBS and soaked in medium overnight. With five repeats per material, scaffolds were transferred to a new 48 well plate for addition of cells.

**Table 1:** Characteristics of scaffolds derived from marine sponges compared to polyurethane sponge

	Marine sponge (%)	PU sponge (%)
Microporosity	33.90 ± 4.66	14.72 ± 9.49
Macroporosity	34.22 ± 7.06	60.73 ± 13.26
Total porosity	67.8 ± 4.145	73.35 ± 2.83

Microporosity was defined as <10 μm diameter [10]. Mean ± SD

### Cell seeding protocol

There is no standard procedure for seeding cells onto a 3D scaffold in culture yet the time for initial cell attachment could be crucial to eventual colonization of the material. For both cell types, two different loading protocols were tested. In the first (method A), 1 × 10^5^ cells in 50 μL of complete medium appropriate to cell type (i.e. DMEM: Ham’s F12 supplemented with 10 % foetal bovine serum and 0.6 mg/mL geneticin [all Gibco,] for hFOBs and DMEM plus supplements for gpBMSCs) were added to each scaffold and incubated for 1 h before an additional 1 mL of medium was added to fill the well. In the second protocol (method B), 1 × 10^5^ cells in 20 μL of complete medium were added to each scaffold. Every 30 min, an additional 20 μL of medium was applied to prevent the materials drying and, after 4 h, the wells were filled with medium.

Cells were fed by complete changes of medium twice a week. During the first medium change at day 4, cell death was established by quantifying the release of lactate dehydrogenase (LDH) into the culture medium using CytoTox 96^®^ non-radioactive assay (Promega, UK) according to the manufacturer’s instructions. After 7 days in culture, medium was removed, cells were washed twice with PBS and then 700 μL of lysis buffer (0.1 % Triton X-100 [Sigma Aldrich, UK] in PBS) was added to each well. The plate was subjected to three cycles of freezing at −80 °C and thawing at 37 °C to lyse all cells. A picogreen assay (Quant-IT™ Picogreen^®^, Molecular Probes, Invitrogen, UK) was then performed on the cell lysate according to manufacturer’s instructions to establish cell number by measuring the amount of dsDNA.

### Cell attachment

Using seeding method B described above, 1 × 10^5^ hFOBs were loaded onto each material with 4–5 repeats per experimental condition. Cell attachment to tissue culture plastic was used as a positive control. Cells were fed by complete change of medium twice a week. After 4, 7 and 14 days in culture, samples were washed ×2 with PBS, transferred to a clean 48 well plate and covered with 700 μL of lysis buffer for preparation of lysates as above. LDH and picogreen assays were performed as above.

Additional scaffolds were prepared for qualitative analysis by SEM and confocal microscopy. Cells (hFOBs or gpBMSCs) were seeded onto 10 × 10 mm materials using method B described above. After 7 days in culture, samples for SEM were fixed in 2 % glutaraldehyde in 0.1 M sodium cacodylate buffer, washed ×2 in sodium cacodylate buffer and dehydrated through graded alcohols. After drying with HMDS overnight, scaffolds were sputter coated with gold and viewed on a scanning electron microscope (JEOL 6500F, JEOL Ltd., Tokyo, Japan). Scaffolds to be viewed by confocal microscopy were also incubated for 7 days after which time viable cells were labeled with fluorescent microspheres (CelLuminate™, Biocompatibles International, UK). CelLuminate™ reagent was added to each well at concentration of 10 % v/v and incubated for 24 h. After washing x3 in PBS, materials were viewed on a confocal laser scanning microscope (Carl Zeiss Image M1, Carl Zeiss Ltd, UK) at an excitation wavelength of 580 nm.

In order to determine the extent of cell penetration through the material, a set of 10 × 10 mm samples were cut in half in cross-section and then placed back together again (in order that they could be split at the end of the experiment for internal analysis of cell distribution) before hFOBs were seeded onto the top of the scaffold as the protocol above. After 4 days, cells were stained with a Live/Dead^®^ Reduced Biohazard Viability Kit (Molecular Probes) according to the manufacturer’s instructions. Following staining, cells were fixed with 4 % glutaraldehyde, washed with PBS and viewed on a confocal laser scanning microscope as described above.

### Cell differentiation

hFOBs were loaded onto scaffolds as previously described and cultured in complete medium for 24 h after which medium was replaced with osteogenic medium (i.e. for hFOBs complete medium plus 50 μM ascorbate-2-phosphate: for gpBMSCs complete medium plus 50 μM ascorbate-2-phosphate, 0.1 μM dexamethasone 10 μM β-glycerophosphate). In addition, hFOBS were cultured at 39 °C, a temperature that restricts proliferation and encourages differentiation of this cell line. After 7 days, four repeats per material were processed for determination of alkaline phosphatase activity and the rest were analysed for gene expression.

Alkaline phosphatase activity was measured in cell lysates produced using the same method as described previously for picogreen assay. Fifty microlitres of each lysate and standard was transferred into a 96 well assay plate. Standards were prepared from p-nitrophenol (Sigma Aldrich) diluted with in lysis buffer (0.1 % Triton X-100 in PBS). 200 μL of substrate buffer (p-Nitrophenylphosphate disodium, Sigma™104, dissolved in 1.5 M 2-Amino-2-methyl-propanol, Sigma™221) was added to each well and incubated for 30 min at room temperature in the dark. Following the addition of 50 μl of stop solution (1 M NaOH) the absorbance was read at 405 nm using a Tecan GENios microplate reader.

Expression of COL1A1 and osteocalcin genes were measured using real-time PCR and compared to the expression of the housekeeping gene, GAPDH. Total RNA was extracted and pooled from five repeats of each material using GenElute (Sigma Aldrich). Briefly, cells were lysed in a 2-mercaptoethanol buffer, diluted with ethanol and passed through an RNA binding column. RNA was eluted and quantified using a NanoDrop™ 1000 spectrophotometer (Thermoscientific from Life Technologies) before being transcribed to cDNA (Transcriptor first strand cDNA synthesis kit, Roche, UK) using the procedure for Anchored-oligo(dT)18 primers. cDNA was stored at −20 °C before use. 2 μL of cDNA was added to 10 μL of mastermix (Fast Start Taqman probes master, Roche), 1 μL of Real-time Ready primer kit for each gene of interest (Roche, UK) and dH_2_O to a final reaction volume of 20 μL. The PCR reaction was performed in a Rotor-Gene Q (Qiagen, UK) at an annealing temperature of 60 °C. Expression levels of all genes were normalized to GAPDH mRNA levels.

### Conditioned medium experiments

In order to establish if there were differences in the chemical dissolution products from the materials that may account for differences in cell response, a conditioned medium experiment was performed.

Each material was soaked in complete hFOB culture medium at 1 g/mL for 14 days. Samples of the conditioned media were sent for analysis by inductively coupled plasma–mass spectrometry (ICP-MS) whereby elemental analysis was performed to determine Ca, P, Mg and Si concentrations using a Perkin Elmer Optical Emission Spectrometer, Optima 4300DV. Prior to analysis the machine was calibrated to 10 ppm with a detection limit of 0.01 mg/L. The remaining conditioned medium was diluted with complete medium to give final concentrations of conditioned media of 1, 0.5, 0.25, 0.1, and 0.01 with complete medium as control.

To evaluate cell response to conditioned media, hFOBs were seeded at 1 × 10^5^ cells/cm^2^ in 96 well plates and allowed to adhere for 1 h at 33 °C and 5 % CO_2_ atmosphere. After this time, the growth medium was removed and 200 μl of each concentration of conditioned media was applied with four repeats. Medium was replaced with the same concentration of conditioned medium after 24 h and twice a week thereafter. A cytotoxicity assay was performed on media removed at day 1 using LDH assay as above. After 14 days in culture, cell lysates were prepared as above and cell number established using picogreen assay.

### Statistics

Differences between groups were analysed using one way ANOVA with Bonferroni’s post hoc testing. Differences to control medium were determined using ANOVA with Dunnett’s post hoc testing. Significance was accepted if *P* < 0.05.

## Results

### Seeding protocol

**Fig. 2:**
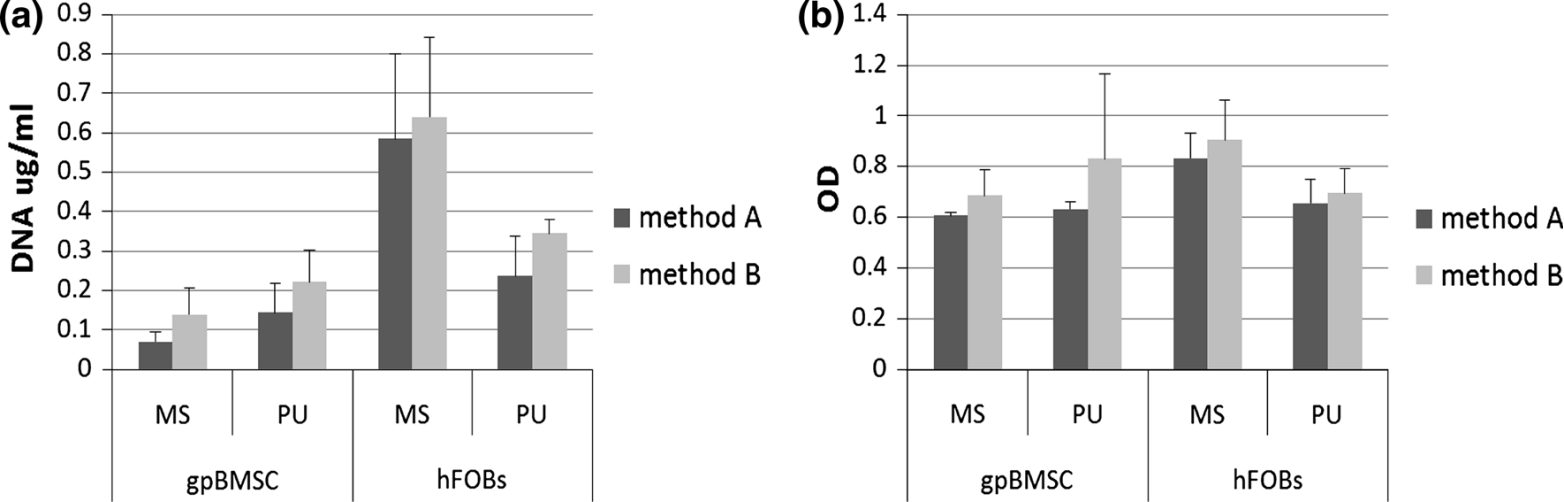
Cell number (**a**) and cell death (**b**) results for each scaffold when incubated with hFOBs and gpBMSCs using two cell seeding protocols. For details of protocols see text. Mean + SD. n = 5. *MS* marine-derived scaffold, *PU* polyurethane-derived scaffold

There was no significant difference in either cell number or cell death between the two different methods used for cell seeding (Fig. 2). As the trend was for slightly higher cell numbers on each scaffold with method B, this method was chosen for the remainder of the experiments.

### Cell attachment

**Fig. 3:**
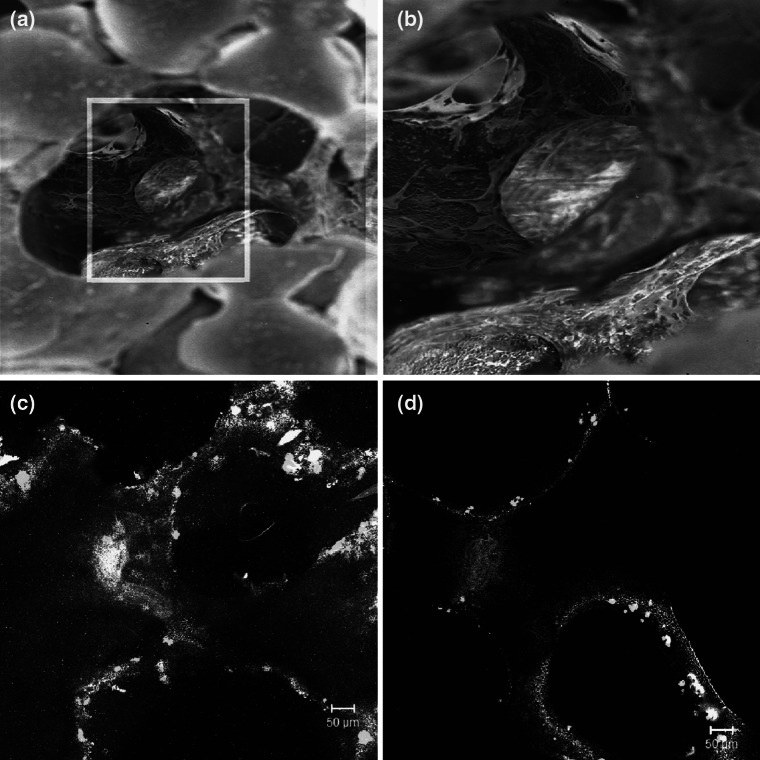
Cells migrating into the pores shown by SEM (**a**, **b**) on PU-derived scaffolds (gpBMSCs) and hFOBs on the inner surface of marine-derived (**c**) and PU-derived (**d**) scaffolds following fluorescent labeling of live cells (*green*). Dead cells would be labeled red but none are visible (Color figure online)

After 7 days in culture both materials supported hFOB and gpBMSC attachment (Fig. 3). SEM showed that the cells were elongated with extensive processes attaching to the material surface (Fig. 3a, b). It was also possible to observe cells deep within the scaffold microstructure by SEM and this was confirmed by the presence of cells on the internal cut surface of both materials showing cells in the centre of the scaffold (Fig. 3c, d).

### hFOB proliferation and differentiation

**Fig. 4:**
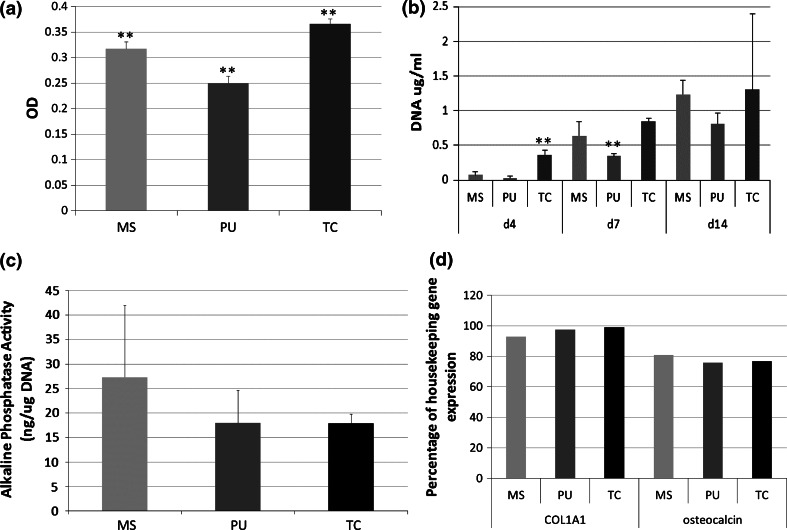
Cytotoxicity (**a**), cell proliferation (**b**) and osteogenic differentiation (**c**, **d**) of hFOBs cultured on each material. Osteogenic differentiation of hFOBs shown by alkaline phosphatase activity normalised to μg of DNA (**c**) and expression of osteogenic genes (**d**) at day 7. Mean + SD. *MS* marine-derived scaffold, *PU* polyurethane- derived scaffold, *TC* tissue culture plastic. **Statistically significantly different from other groups at the same time point (*P* < 0.01)

Both scaffolds had reduced cytotoxicity levels compared to cells grown on tissue culture plastic (Fig. 4a) with cytotoxicity on PU-derived scaffolds also significantly lower than marine-derived scaffolds. As small amounts of LDH can be released from viable cells, some of these differences may be accounted for by differences in cell number. Importantly, cell death levels across all experimental conditions was relatively low. hFOB proliferation on both scaffolds increased with time (Fig. 4b). At d4 cell numbers were lower than that on tissue culture (TC) plastic control, this was reversed by 7 days for marine-derived materials and by d14 for PU-derived materials at which time there was no significant difference between groups. Scaffold type did not have any effect on the osteogenic differentiation of the cells as measured by alkaline phosphatase activity (Fig. 4c) and collagen 1A1 and osteocalcin gene expression (Fig. 4d).

### Conditioned medium experiments

**Fig. 5:**
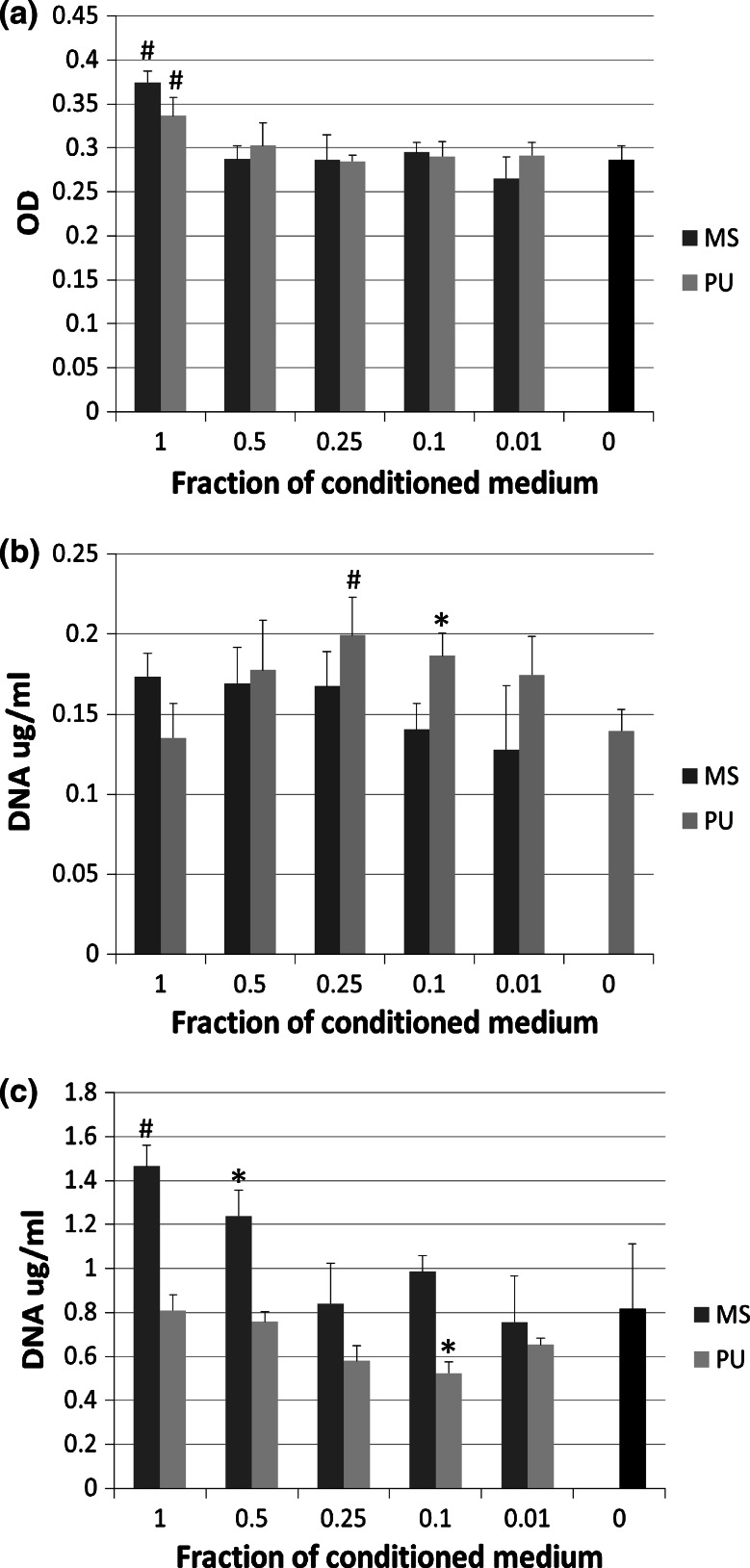
hFOB cell death at day 1 (**a**) and cell number after 1 day (**b**) and 14 days (**c**) in culture with decreasing concentrations of conditioned medium. *MS* marine-derived scaffold, *PU* polyurethane-derived scaffold. *Bars* indicate mean + SD. n = 4. ^#^Statistically different from 0 % control group at *P* < 0.01 and *P < 0.05

There was an increase in cell death when hFOBs were cultured in 100 % conditioned medium (CM) from all four testing conditions (*P* < 0.001, Fig. 5a). As the CM concentration was diluted however, there was no difference in LDH release compared to control medium. There were no significant differences between the CM derived from the PU-derived scaffold compared to the marine-derived scaffold.

Results from the picogreen assay show that there was a trend towards an increase in cell number with increasing concentration of CM from marine-derived scaffolds at d1 but these were not significantly different from control (*P* = 0.055, Fig. 5b). In contrast, cell number showed a bell-shape curve in response to fractions of CM from PU-derived scaffolds at d1 which was significantly different (*P* = 0.004 by ANOVA). Both the 0.1 and the 0.25 fractions were significantly higher than control media (0.035 and 0.006 respectively), suggesting there is an optimum concentration of soluble factors in this medium. By day 14, this trend had reversed and there was now a significant reduction in cell number with the 0.1 fraction of CM from PU-derived scaffolds compared to control (*P* = 0.015, Fig. 5c). The earlier trend seen in response to CM from marine-derived materials had continued and now reached statistical significance (*P* < 0.001 by ANOVA); post hoc testing showed this was significant at the highest two fractions compared to control (*P* = 0.018 and *P* = 0.001).

ICP-MS analysis showed a number of differences between the CM from marine-derived and PU-derived scaffolds with an almost four-fold increase in Ca concentration, a ten-fold decrease in P and increased amounts of Si in the marine-derived media extracts (Table 2). This reflects the presence of Si found in marine-derived scaffolds by EDX (Fig. 1).

**Table 2:** Elemental analysis of concentrated (100 %) conditioned media extracts from both scaffolds by inductively-coupled plasma-mass spectroscopy

	Marine derived	Synthetic derived
Ca (mg/L)	193	59.1
P (mg/L)	3.44	30.1
Si (mg/L)	2.08	0.37
Mg (mg/L)	74.9	96.7

## Discussion

The hypothesis was that the naturally occurring, unique pore architecture of the marine-derived scaffolds would provide an enhanced environment for cell growth when compared to a synthetic polymer derived scaffold. The results showed that, although cells demonstrated good attachment to both scaffold types, with an elongated morphology and extensive processes, they proliferated faster and colonized the marine-derived scaffold more quickly than the PU-derived scaffold. They were found at the centre of both scaffolds, remaining viable to at least 14 days suggesting either that the cells were able to migrate into the scaffolds or that they percolated significant depths during seeding. These results confirm the promising early qualitative results which suggested that osteoblast-like cells and endothelial cells attached better to the marine-derived material [16]. The ability of the cells to differentiate as shown by alkaline phosphatase activity and expression of collagen IAI and osteocalcin genes was not enhanced but, encouragingly, neither was it inhibited by the scaffolds and osteoblast differentiation was maintained at levels similar to the control. These results are similar to those reported by others who found that osteoblast proliferation and migration/penetration into scaffolds was enhanced by pore size but differentiation or mineralization was unaffected [17, 18].

The importance of pore characteristics in bone graft materials has been well described [8, 9, 11, 19, 20]. Key features include a range of pore sizes on both the micro and macro scale, a degree of interconnectivity of the pores, allowing fluid diffusion and cell migration through the material, and finally a degree of tortuosity (scaffolds in which the route through the material is straightforward tend to reduce cell capture) [21]. The marine-derived hydroxyapatite scaffold provides all of these features with 33.9 % microporosity, 34.2 % macroporosity, 99.9 % interconnectivity and permeability similar to that of human bone [16] and others have demonstrated optimum oxygen diffusivity in scaffolds derived from marine sponge templates compared to polyurethane foam [22].

However, the enhanced cell proliferation reported within this study may not just be related to the architecture of marine-derived scaffolds. EDX analysis showed that marine-derived scaffolds contained Si which was not present within the PU-derived scaffold and both scaffold types contained similar amounts of Mg. Secondary ions, which were once classified as impurities, have now been recognized as important for bone repair, particularly as the mineral phase of bone is non-stoichiometric HA and contains a number of minor ions such as carbonate (CO_3_^2−^), magnesium (Mg^2+^) and silicate (SiO_4_^4−^). This has led to interest in ion-substituted ceramic materials as bone graft materials [23].

In order to differentiate the effects of scaffold architecture from the effects of scaffold chemistry, a conditioned medium experiment was performed. CM derived from both scaffold types reflected the EDX elemental analysis with confirmed presence of Mg in both and the addition of Si in CM from the marine-derived scaffolds. Although the sponge species used for fabrication of the scaffolds does not contain spicules, it is believed that harvesting methods adopted when collecting from the Mediterranean Sea led to contamination with silicate spicules derived from other species which then become incorporated into the HA scaffold during sintering. In addition, CM from the marine-derived scaffolds contained more than three times the amount of Ca and a fraction of the phosphorus levels compared to PU-derived scaffolds.

Results from the CM experiments suggest that cell proliferation was increased initially (at day 1) by optimal concentrations of CM from PU-derived scaffolds, with the highest and lowest concentrations reducing proliferation. Although this effect was later lost by day 14, this early acceleration is most likely to be due to the presence of Mg ions. Magnesium has been reported to enhance osteoblast adhesion, increase angiogenesis in porous structures [24], increase bone formation [25] and increase bioresorption [26, 27]. Crucially, in vitro, several papers have reported that high and low concentrations of Mg are inhibitory to cell proliferation and/or cytotoxic but optimal concentrations can enhance osteoblast proliferation and mineralization [28, 29]. The findings of the current study support this but would suggest that this initial increase is transitory.

CM from marine-derived materials, in contrast, did not show the same bell-shape curve in cell proliferation with decreasing concentration at day 1, despite similar concentrations of Mg as measured by ICP. This could mean that the effects of the PU-derived CM are not related to Mg content or, more likely, that the presence of Si or increased Ca levels in the marine-derived extracts is masking the Mg effects. Indeed, at day 14 the highest cell numbers were seen with the highest concentrations of CM from marine-derived materials. Si-based glasses (Bioglass^®^) and Si-substituted calcium phosphates (CaP) have been widely studied in bone repair [30, 31]. Bioglass^®^ has been shown to have proangiogenic properties [32–35], to bond directly to bone [36], to support osteoblast attachment and differentiation [37–39] and to enhance bone repair [31]. Similar claims have been made for Si-substituted CaP ceramics [40–42] and for other silica-based biomaterials [23, 43, 44] however the mechanism of action is unknown and there is some controversy over whether it is a direct effect of the substituted ion or an indirect effect caused by changes to the physical characteristics of the material [23, 30]. There is growing evidence for a direct effect of Si on the pathways of bone formation however [45, 46] as, for example, Beck et al. reported that silica nanoparticles mediated bone formation by suppression of NF-κB [43], and others reported that biosilica enhanced the expression of osteoprotegerin (OPG) and runt-related transcription factor 2 (Runx-2) [47].

CM from marine-derived extracts also had increased Ca levels and decreased P levels. Quite why the phosphate disappeared in the marine-derived CM is unclear but others have reported similar phenomena when producing CM from bioactive glasses [48]. The authors attributed this to surface reactions during ionic dissolution and suggested that it could contribute to delayed differentiation of osteogenic cells as there was no source of phosphate for mineralization. There have not been any reports of an increase in proliferation associated with low phosphate levels so the effects seen here are more likely to be due to other differences such as the Si and Ca levels. An increase in Ca ion concentration to five times standard culture medium has been shown to be associated with an increase in rat bone marrow stromal cell proliferation, migration and osteogenic differentiation, and increasing to levels above this were found to inhibit the response [49]. Levels in the CM from marine-derived materials used in this study were 2.5 times higher than standard culture medium and that from PU-derived materials were slightly lower than standard medium (59 mg/L compared to 76 mg/L) so the difference in Ca concentration could also be contributing to the increase in cell proliferation reported here. In addition, there is evidence that a sudden change in Ca concentration, either higher or lower, can induce an increase in intracellular Ca levels in osteoblasts [50], therefore, not only the absolute increase in marine-derived CM but also the sudden switch to this media, may have induced a response in the cells.

Ion substitution is generally considered a positive addition to a ceramic material to enhance bone repair and marine organisms are a rich source of these ions which can become incorporated into a biomaterial when converting the base mineral into CaP. For example, the aragonite skeleton of cuttlefish can be converted to carbonated hydroxyapatite [51–53] and two species of sea urchins rich in Mg^2+^ ions were found to produce Mg-substituted β-tricalcium phosphate during the conversion process [54]. We have shown that, even when the marine organism is simply used as a preform, biogenic ions are still incorporated into the end product and that these could have a beneficial effect on the ability of the resulting scaffold to support bone formation.

## Conclusion

Three dimensional porous constructs have been manufactured that support osteoblast attachment, proliferation and differentiation but significantly more cells were seen on marine-derived scaffolds using the sponge, *Spongia agarcina*, as a template. This could be related both to the physical and chemical characteristics of the material, with optimum pore characteristics in addition to a biological stimulus from the presence of Si ions. Further in vivo tests in orthotopic models are required but this marine-derived scaffold shows promise for applications in bone tissue engineering.
